# Conservancy of mAb Epitopes in Ebolavirus Glycoproteins of Previous and 2014 Outbreaks

**DOI:** 10.1371/currents.outbreaks.f1a7028a13ce1c5f0bdbb4b0cc0b919b

**Published:** 2014-11-03

**Authors:** Julia Ponomarenko, Kerrie Vaughan, Alessandro Sette, Sebastian Maurer-Stroh

**Affiliations:** University of California San Diego, La Jolla, California, USA; La Jolla Institute, La Jolla, California, USA; La Jolla Institute, La Jolla, California, USA; Bioinformatics Institute (BII), A*STAR, Singapore

**Keywords:** disease outbreak, ebola, ebolavirus, EBOV, epitope, mAb

## Abstract

Background: Several monoclonal antibodies (mAb) are being evaluated as treatment options for the current 2014 Ebola outbreak. But they were derived from and tested for protection against the older 1976 Mayinga or 1995 Kikwit Zaire Ebolaviruses (EBOV). The EBOV sequences reported for the current outbreak contain several mutations whose significance remained to be established. Methods: We analyzed sequence and structural conservation of the Ebolavirus glycoprotein (GP) epitopes for all experimentally identified protective mAbs published to date. Results: The conservancy analysis of protective mAb epitopes in the Ebolavirus glycoprotein sequences spanning all Ebola virus lineages since 1976 showed that conservancy within the Zaire EBOV lineage was high, with only one immunodominant epitope of mAb 13F6-1-2 acquiring two novel mutations in the 2014 outbreak that might potentially change the antibody specificity and neutralization activity. However, the conservation to other Ebola viruses was unexpectedly low. Conclusion: Low conservancy of Zaire EBOV mAb epitopes to other EBOV lineages suggests that these epitopes are not indispensable for viral fitness, and that alternative mAbs could be developed to broadly target all EBOV. However, average percent sequence identity of the epitopes for mAbs used in current cocktails to the Zaire EBOV is high with only one epitope differing in the 2014 outbreak. These data bode well for general usefulness of these antibodies in the context of the current outbreak.

## Introduction

Gire *et al.* characterized the sequence diversity within the 2014 Ebola virus (EBOV) outbreak^1^ and identified several novel mutations. One effect these mutations could have is to alter epitope regions that could be critical for the current situation as drug cocktails comprising monoclonal antibodies (mAbs) are being tested for Ebola disease treatment.

As of to date, six monoclonal antibodies (mAb) comprising the three cocktails, ZMab, ZMapp, and MB-003, have being evaluated as treatment options for Ebola^2^^,^ 3 ^,^ 4 ^,^ 5 ^,^ 6 ^,^ 7 ^,^ 8 . These mAbs were however derived from and tested for protection against the older 1976 Mayinga or 1995 Kikwit Zaire Ebolaviruses. As the new Ebola virus strains, which have emerged in the current 2014 outbreak, are yet to be tested in animal models, it is imperative to computationally assess the conservancy of these and other known protective mAb epitopes across all Ebola viruses.

## Methods

Data on EBOV-related mAb epitopes was obtained from the IEDB 9 , which contains only experimentally identified epitopes. Predicted epitopes were not considered in this study. For the data considered herein (Table 1 and Figure 1), epitopes were defined within 14 different papers using common methodologies including, the use of synthetic peptides immobilized on membranes in competition with soluble peptides in competition ELISA (6D8, 13F6, and 13C6), Ebola virus-like particles transfected with GP protein truncations/deletions tested by ELISA, western blot, immunofluorescence assay and immunoprecipitation (1H3, 4G7 and 2G4), X-ray crystallography (13F6 and KZ52) and loss of reactivity to escape mutants (133/3.16 and 226/8.1).

Sequences of non-identical full length Ebolavirus glycoproteins were obtained from Genbank on Aug 28, 2014, and split phylogenetically into 5 groups representing the main lineages plus the 2014 sequences as separate group. Sequences were aligned using the MAFFT L-INS-I^10^ algorithm. Figure 1 with alignment was created with Jalview^11^.

The epitopes were mapped on the GP sequence based on epitope residue positions provided in the IEDB; the specific strain provided for each epitope in the IEDB allows for unambiguous residue mapping.

Residue conservation was calculated for the GP protein, using the ConSurf^12^ website and the non-redundant EBOV alignment. Figure 2 was created with Yasara^13^.

FoldX^14^ was used with 5 repetitions to calculate the change of the free energy, ΔΔG, upon the T411A mutation in [PDB: 2QHR] after energy minimization.

## Results

As of September, 2014, analysis of the Ebola GP protein epitopes reported in the Immune Epitope Database (IEDB) and identified using assays demonstrating *in vitro *correlates of protection (virus neutralization) or *in vivo *survival assays, so-called ‘functional epitopes,’ revealed ten epitopes for nine mAbs (**Fig. 1**). Four of these epitopes were discontinuous, with two, 16F6 and KZ52, obtained from the X-ray structures of the GP-antibody complexes, and the other two, 133/3.16 and 226/8.1, from the experiments using escape mutants. Only one of the nine mAbs was derived from a human survivor of the Ebola hemorrhagic fever (EHF) of the 1995 Kikwit outbreak (mAb KZ52). All other mAbs were either obtained from a murine model or an engineered Fab derived from a display library.

**Functional EBOV-related mAb epitopes reported in the IEDB.**
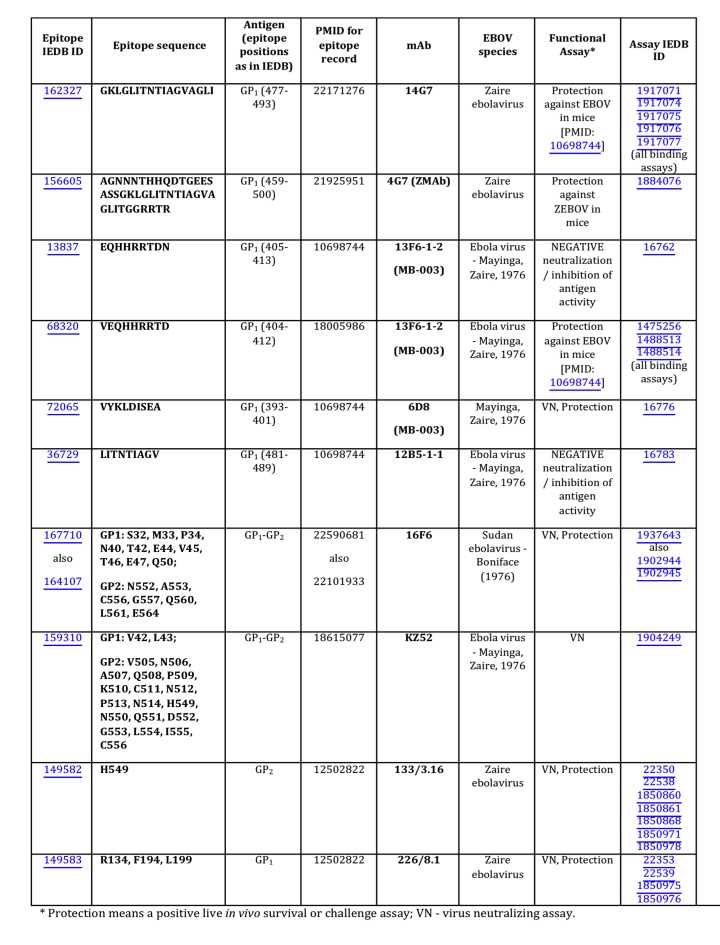

The mapping of the functional mAb epitopes to the alignment of the 42 non-identical sequences of the GP proteins from all EBOV is shown in **Figure 2**. Three of these epitopes, 6D8, 13F6, and 4G7, are mapped to the heavily glycosylated mucin-like domain of the Ebola GP protein spanning the GP1 residues 313 to 501. This domain is unique to GP, includes the GP surface available for host interaction, and is not shared with sGP (a dimeric, soluble glycoprotein that shed abundantly by infected cells), thus making it a desirable target for the Ebola immunotherapeutics^15^ . This domain was not solved in the reported crystal structures of the GP proteins [PDB: 3CSY, 3VE0, 3S88], due to its increased flexibility^16^.

**Ebolavirus sequence conservation of known protective mAb epitopes.**
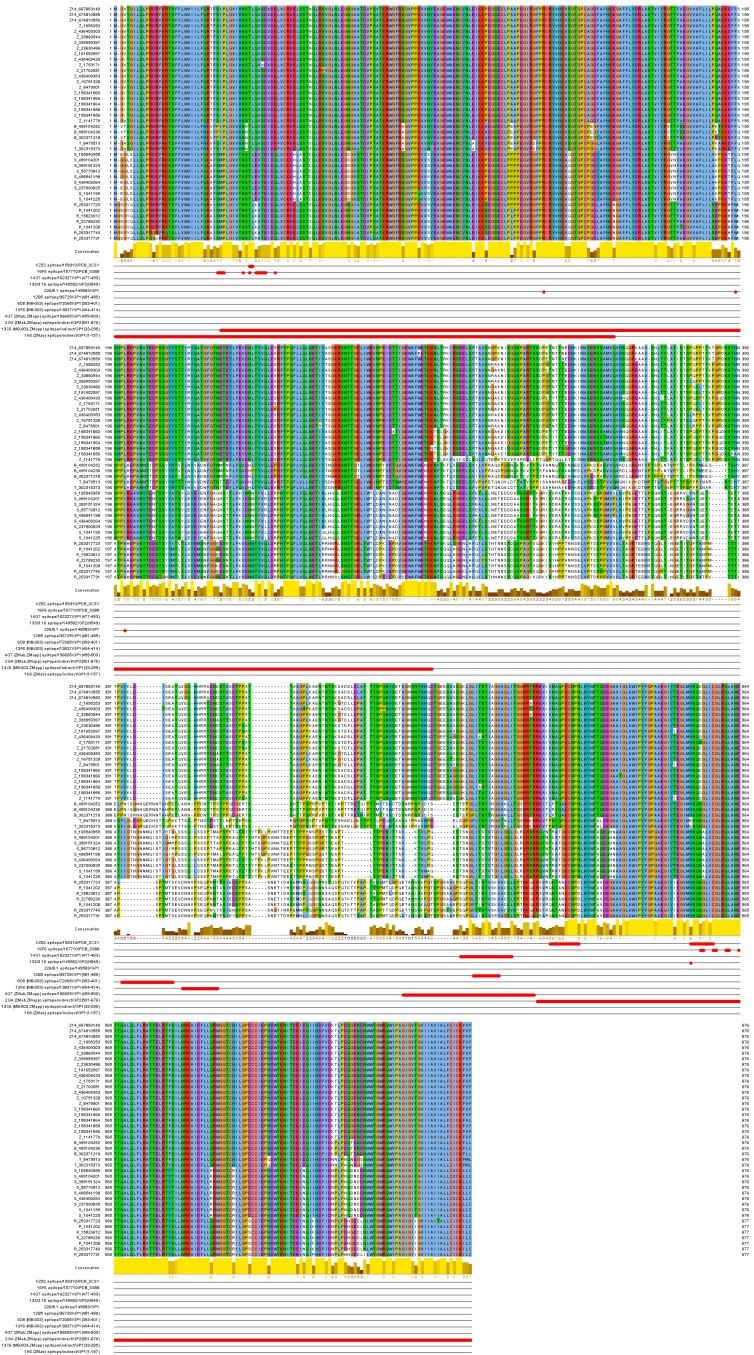
Sequences are labeled with group symbol (Z14 and Z…Zaire, B…Bundibugyo, T…Tai Forest, R…Reston, S…Sudan) and NCBI GenInfo identifier. Epitopes are displayed in rows below labeled with mAb name, IEDB identifier and protein region position or PDB code for conformational epitopes.

The sequence conservancy of six of the functional epitopes for the most tested protective mAb cocktails, ZMab, ZMapp, and MB-003, were evaluated for all Ebola viruses and is provided in **Figure 3**. It can be seen that conservancy in other than Zaire EBOV – that is, Bundibugyo, Tai Forest, Reston, and Sudan viruses – varied from 0 to 90%, with the 2G4 epitope being the most conservative (above 78%) and the 6D8 and 13F6 epitopes, the least conservative (below 23%). For the Zaire EBOV, average sequence identity of all but one epitope was above 98% in both previous and current outbreaks.

**Sequence conservancy of epitopes from the most tested protective mAb cocktails.**
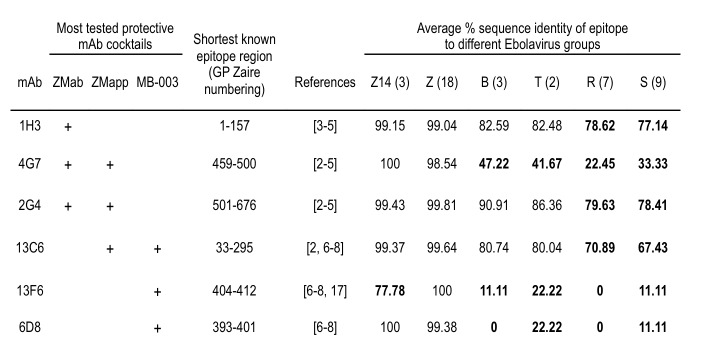
Epitope sequences used to derive mAbs were compared to the complete non-redundant set of available sequences from different Ebolavirus groups (Z14 and Z…Zaire, B…Bundibugyo, T…Tai Forest, R…Reston, S…Sudan; group size in parenthesis). Average % sequence identity of epitopes used in current mAb cocktails to the Zaire lineage is high and only one epitope differs in the current outbreak. Conservancy to other lineages is mostly below 80% (indicated in bold).

This epitope is the immunodominant epitope 404-VEQHHRRTDND-414 of the GP1 protein recognized by mAb 13F6-1-2 (part of mAb cocktail MB-003). It was identical in all Zaire EBOV since 1976 and was considered a good target for vaccine immunotherapeutic design, as it is immunodominant in a murine model, located in the mucine-like domain, provides protection against viral challenge, and does not adopt any secondary structure or posttranslational modifications that would be difficult to achieve in synthetic or recombinant systems^17^.

However, this epitope acquired two novel mutations, namely E405G and T411A that are present in all sequences of the 2014 outbreak. The crystal structure of mAb 13F6-1-2 in complex with this peptidic epitope^17^ [PDB: 2QHR] shows that while the E405G mutation would unlikely have an effect on the antibody binding, the T411A mutation would disrupt a tight hydrogen bond (2.7 Å) between the Thr411 Oγ side-chain atom of the peptide to the Asp33 Oδ1 side-chain atom of the antibody heavy chain, potentially changing the antibody specificity and neutralization activity. Indeed, estimating the effect of this mutation in the 2QHR structure with FoldX predicts the destabilization of the peptide-antibody interaction by 1.24 kcal/mol (energy changes more than +- 0.5 kcal/mol are considered significant effects in FoldX).

By mapping the sequence conservation from the non-redundant EBOV alignment to the GP trimer structure [PDB: 3CSY] one can visualize the distribution of conserved and variable regions on the surface (**Fig. 4A**). Further considering escape mutants and antibody complexes with the GP trimer [PDB: 3CSY, 3VE0, 3S88], suggests a consensus binding region comprised by the conformational epitope (**Fig. 4B**) where mAb 2G4 (part of ZMab and ZMapp) is expected to bind^3^. At its center is a surface-accessible linear peptide (the N-terminus of GP2) that is not well conserved among different EBOVs which may affect inter-lineage cross-protection of antibodies recognizing this region. However, within the Zaire EBOV, there is only one mutation consistently present in the 2014 outbreak, A503V, which is structurally at the border of the region and is predicted to have limited effects on the antibody activity.

**Ebolavirus structure conservation of known protective mAb epitopes.**
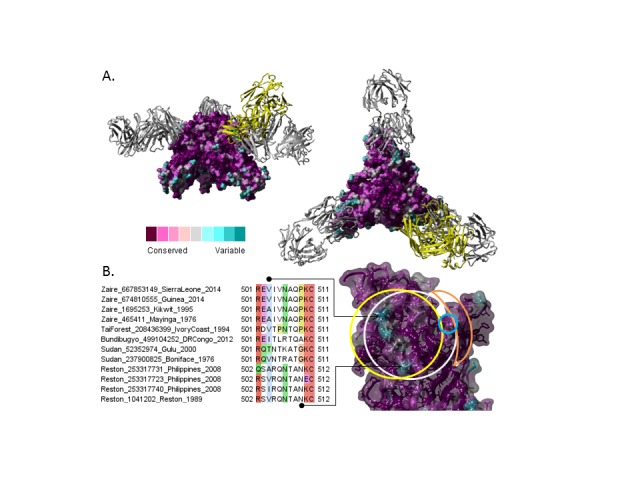
**A.** Ebolavirus glycoprotein trimer [PDB: 3CSY] in complex with monoclonal antibodies KZ52 (gray) and 16F6 (yellow, superimposed from [PDB: 3VE0]). Glycoprotein surface colored by increasing conservation from cyan to purple over the non-redundant EBOV alignment described in Figure 1. **B.** Detailed view of mapped binding sites for mABs KZ52 (gray), 16FG (yellow), 133/3.16 (blue) and 226/8.1 (orange) with sequence alignment of corresponding variable region. Sequence names formatted as Lineage_NCBI-GI_Location_Year.

## Conclusion

The presented analysis of mAb epitopes is novel in respect to a complete conservancy analysis spanning all Ebola virus lineages since 1976 until now and considers the GP protein not only at the sequence but also the structural level as several of the epitopes are conformational. Our two main findings from the analysis are the following: (1) among 6 epitopes targeted by different mAb cocktails only one seems to be significantly different in the current outbreak compared to the earlier ones; and (2) the conformational epitope of GP2 targeted by multiple mAbs includes a region that is not well conserved among the different lineages, indicating that cross-protection from non-Zaire outbreaks to the current one may be limited.

The provided sequence and structural mapping of the epitopes also allows closely following the further evolution of these sites within the outbreak as new sequences become available and hence should be a useful contribution to the current joint global efforts to tackle the serious outbreak.
